# Supplementary material for: Escape from thymic deletion and anti-leukemic effects of T cells specific for hematopoietic cell-restricted antigen
Source: Nat Commun. 2018 Jan 15;9:225. doi: 10.1038/s41467-017-02665-z (PMC5768767; doi:10.1038/s41467-017-02665-z)
Supplement: Supplementary file 1 — Supplementary information [file 41467_2017_2665_MOESM1_ESM.pdf]

Supplementary Figure 1

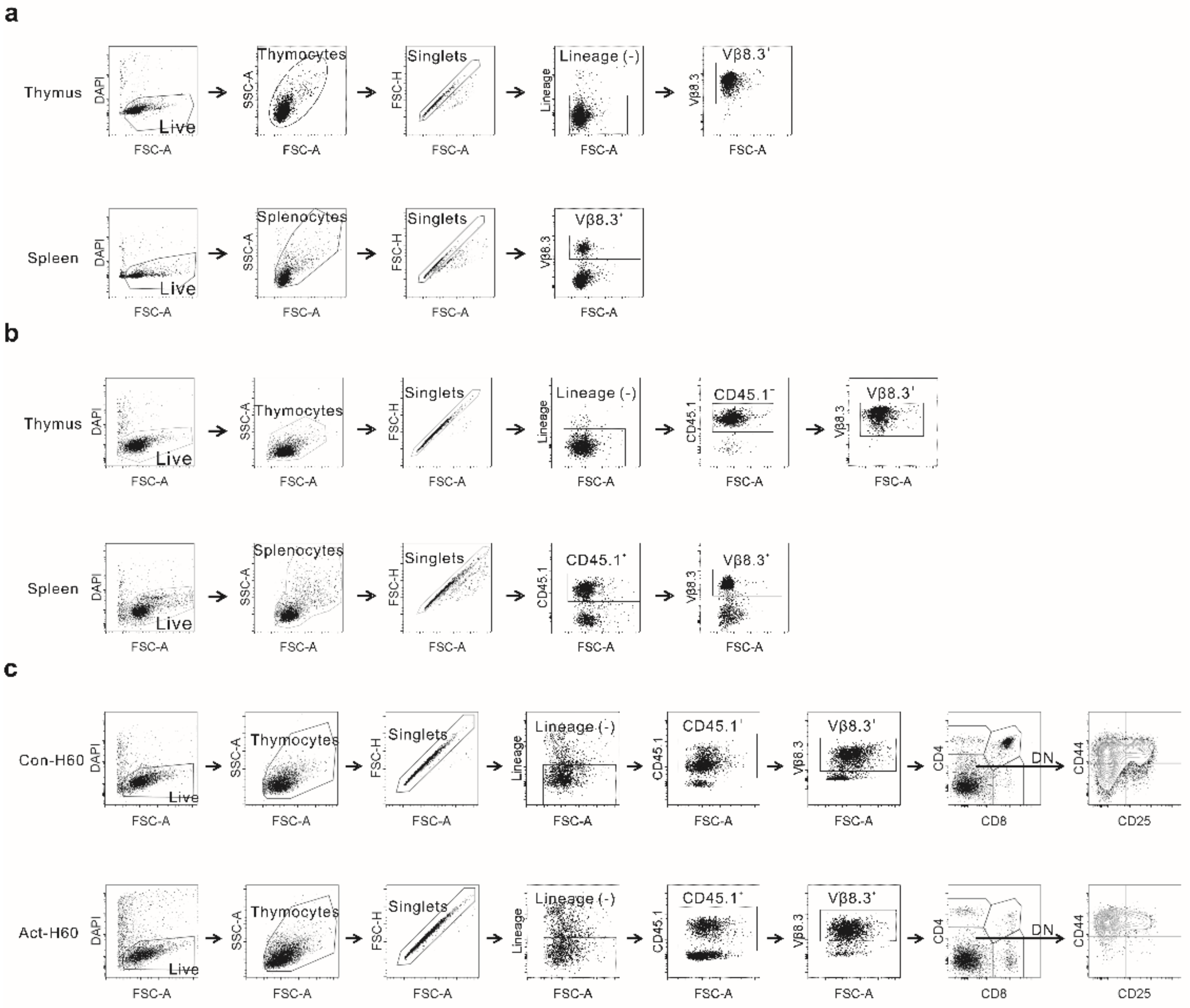

Supplementary Figure 1. Gating strategy for analysis of thymocytes and splenocytes

Dead cells, ascertained by DAPI staining, were removed from the analysis. Thymocytes and splenocytes from (**a**) J15 F1 mice, (**b**) J15→B6 BMT recipients, and (**c**) J15→Con-H60 or J15→Act-H60 BMT recipients were selected using FSC-A (forward scatter area) and SSC-A (side scatter area). Doublets were removed using FSC-A and FSC-H (forward scatter height). Thymocytes were gated in Lin-eFluor710$^-$, CD45.1-eFluor450$^+$, and Vβ8.3-FITC$^+$ cells and were then analyzed for the surface expression of protein markers, including CD4 (PE.Cy5), CD8 (APC.Cy7), CD44 (PE.Cy7), CD25 (allophycocyanin), and CD5 (PE). Splenocytes were gated in Vβ8.3$^+$ cells and then analyzed based on CD4 and CD8 expression. (**a**) is related to Fig. 1, (**b**) to Fig. 2, Fig. 4a, Fig. 4c, Fig. 4g, Fig. 4h, and Fig. 5, and (**c**) to Fig. 3, and Fig. 4d, Fig. 4e, Fig. 4i, and Fig. 4j.

Supplementary Figure 2

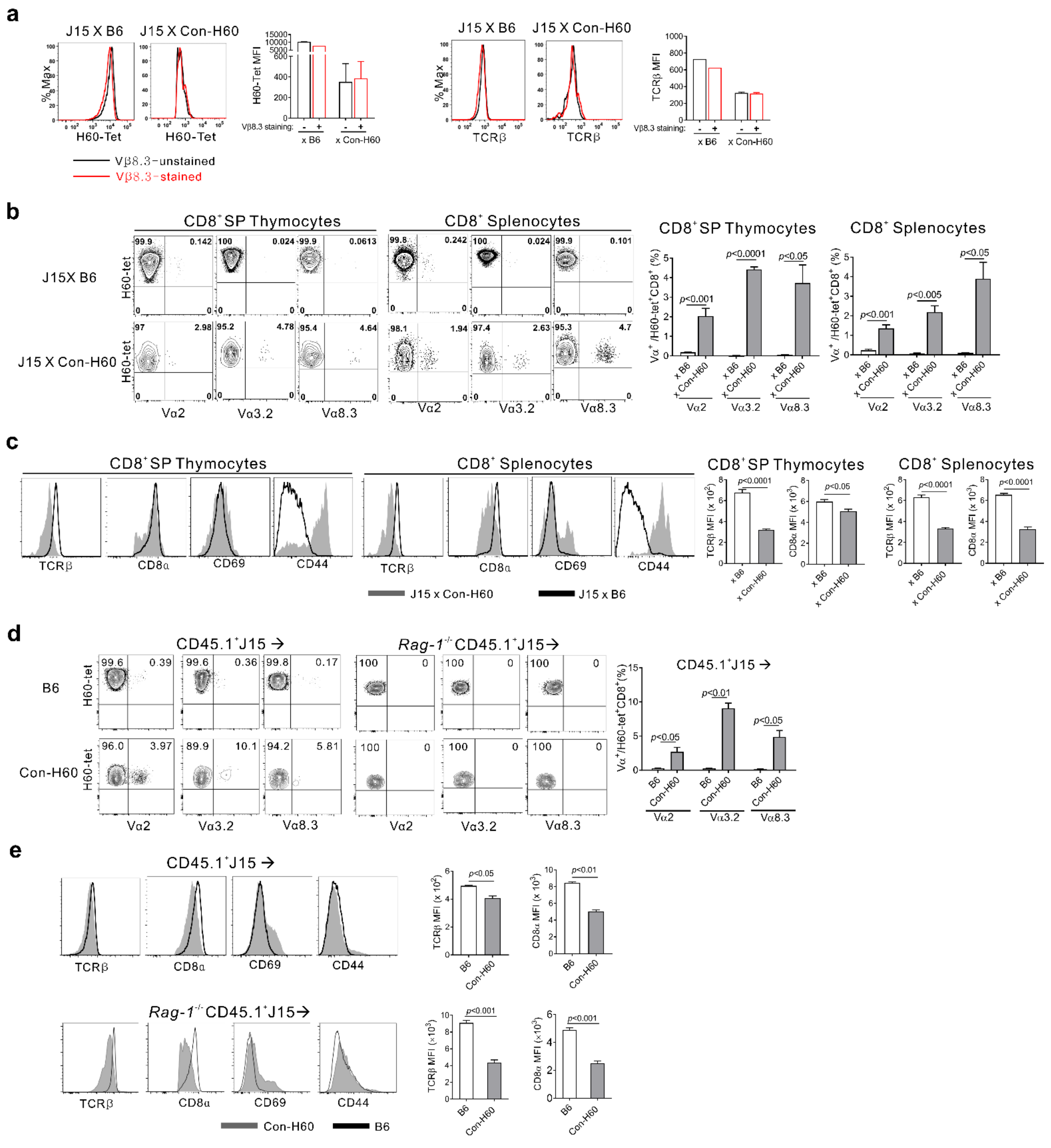

Supplementary Figure 2. Characterization of J15 CD8$^+$ cells generated in Con-H60 hosts.

(**a**) H60-tetramer or TCRβ staining in the presence and absence of anti-TCR Vβ 8.3 staining of CD8$^+$ SP thymocyte generated in [J15 X Con-H60] F1 and [J15 X B6] F1 mice. Representative single histograms show

FACS data on the levels of H60-tetramer-PE staining (left) or TCRβ-APC staining (right) of [J15 X Con-H60] F1 cells and [J15 X B6] F1 cells in the presence (red line) and absence (black line) of staining with TCR Vβ 8.3-FITC. MFI values of H60-tetramer staining and TCRβ-staining are plotted **(b**) Flow cytometric analysis of the expression of endogenous TCRα chains by $CD8^+$ SP thymocytes and splenocytes generated in [J15 X Con-H60] F1 and [J15 X B6] F1 mice. Representative Vα2-APC-Cy7, Vα3.2-APC, or Vα8.3-FITC by H60-tetramer-PE/APC FACS profiles of H60-tetramer$^+$ CD8 T cells are shown. Percentages of Vα2, Vα3.2, and Vα8.3-positive populations among H60 tetramer$^+$ $CD8^+$ cells are plotted. (**c**) Surface expression levels of TCRβ, CD8α, CD69, and CD44 on $CD8^+$ SP thymocyts and splenocytes generated in [J15 X Con-H60] F1 and [J15 X B6] F1 mice. Single histograms show FACS data on the level of the molecules on [J15 X Con-H60] F1 cells (gray filled line) and [J15 X B6] F1 cells (solid line). MFI values of TCRβ and CD8 staining are plotted. (**d**) Expression of endogenous TCRα chains by $CD8^+$ SP J15 thymocytes developed in B6 and Con-H60 recipients of *Rag-1*$^{+/+}$J15 BM or *Rag-1*$^{-/-}$J15 BM. Data are processed as described for (**b**). (**e**) Flow cytometric analysis of surface levels of TCRβ, CD8, CD69, and CD44 on $CD8^+$ SP thymocytes from Con-H60 and B6 recipients of *Rag-1*$^{+/+}$J15 BM or *Rag-1*$^{-/-}$J15 BM. Data are processed as described for (**c**). Data (**a-e**) are from at least three independent experiments (n=2/group/experiment) and are presented as means ± s.e.m. *P* values were generated by Student's t-test.

Supplementary Figure 3

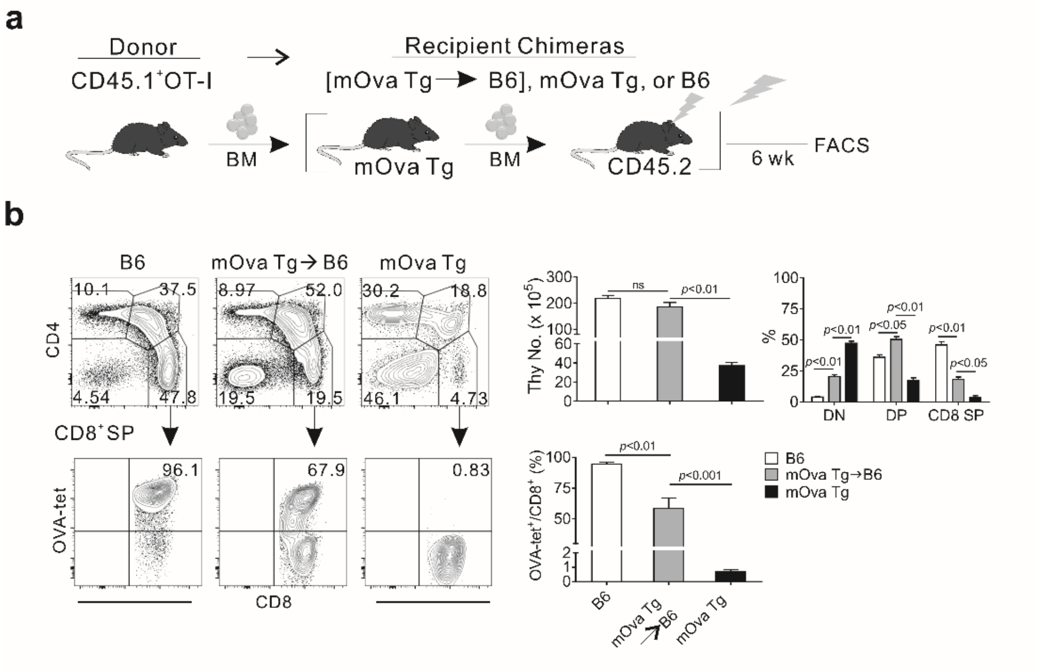

Supplementary Figure 3. Incomplete negative selection of OT-1 CD8 T cells specific for hematopoietic Ova in BMT recipients

(**a**) Schematic illustration showing the experimental design. TCD-BM cells (5×10^6) from mOva Tg mice were transplanted into lethally irradiated B6 mice, mOva →B6. After six weeks, 5×10^6 TCD-BM cells from the CD45.1$^+$ OT-I cells were transplanted into lethally irradiated B6, [mOva Tg→B6], or mOva Tg mice. (**b**) Thymic profiles of the three different BMT recipients. CD45.1$^+$Vα2$^+$ donor BM-derived thymocytes were analyzed at 6 weeks post-BM transplantation. Representative FACS data of the CD4-PE.Cy5 by CD8-APC.Cy7 staining in CD45.1$^+$ Vα2$^+$ thymocytes and of OVA-tetramer-staining in CD8 SP thymocytes are shown. Numbers of total thymocytes and percentages of cells at each thymic developmental stage; OVA-tetramer-binding cell frequencies in CD8 SP thymocytes are plotted as bar graphs. Data (**b**) are representative of three independent experiments (n=2/group/experiment) and presented as means ± s.e.m. *P* values were generated by Student's t-test.

Supplementary Figure 4

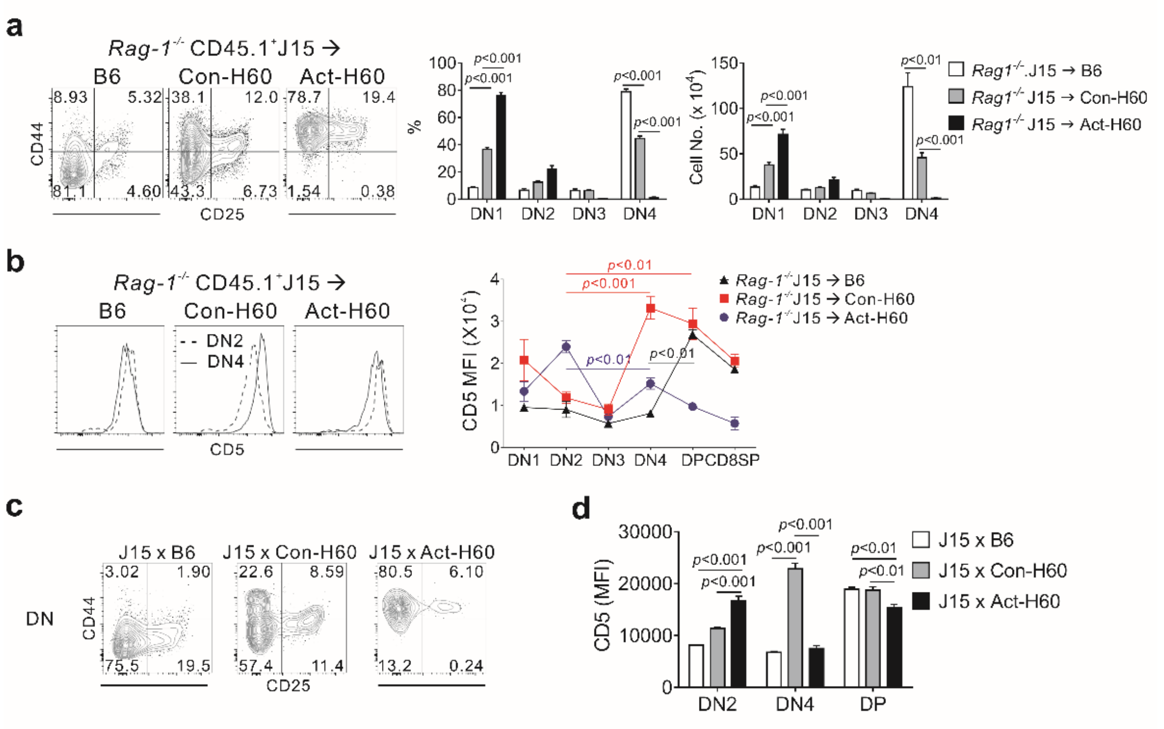

Supplementary Figure 4. Delayed thymic negative selection of J15 T cells in Con-H60 recipients of *Rag-1*$^{-/-}$J15 BMT and J15 x Con-H60 mice

(**a–b**) DN profiles of *Rag-1*$^{-/-}$CD45.1^{+}J15 BMT recipients. (**a**) Representative FACS data of CD44-PE.Cy7 and CD25-allophycocyanin profiles are shown. Values in the FACS data indicate the percentages of each quadrant fraction in the DN cells. These percentages and the corresponding cell numbers are plotted as bar graphs. DN1, DN2, DN3, and DN4 cells indicate the CD44^{+}CD25^{-}, CD44^{+}CD25^{+}, CD44^{-}CD25^{+}, and CD44^{-}CD25^{-} quadrants, respectively. (**b**) Histograms depicting the surface expression of CD5 on DN2 and DN4 thymocytes (left) and MFI values of CD5-PE staining along the thymic development stages of *Rag-1*$^{-/-}$CD45.1^{+}J15 in BMT recipients. (**c–d**) DN profiles of thymocytes from F1 progenies. (**c**) Representative FACS data of CD44-PE.Cy7 and CD25-allophycocyanin profiles and (**d**) bar graph of MFI values of CD5 staining are shown. Data (**a–d**) represent more than three independent experiments (n=3/group/experiment) and are presented as means ± s.e.m. *P* values were generated by Student's t-test.

Supplementary Figure 5

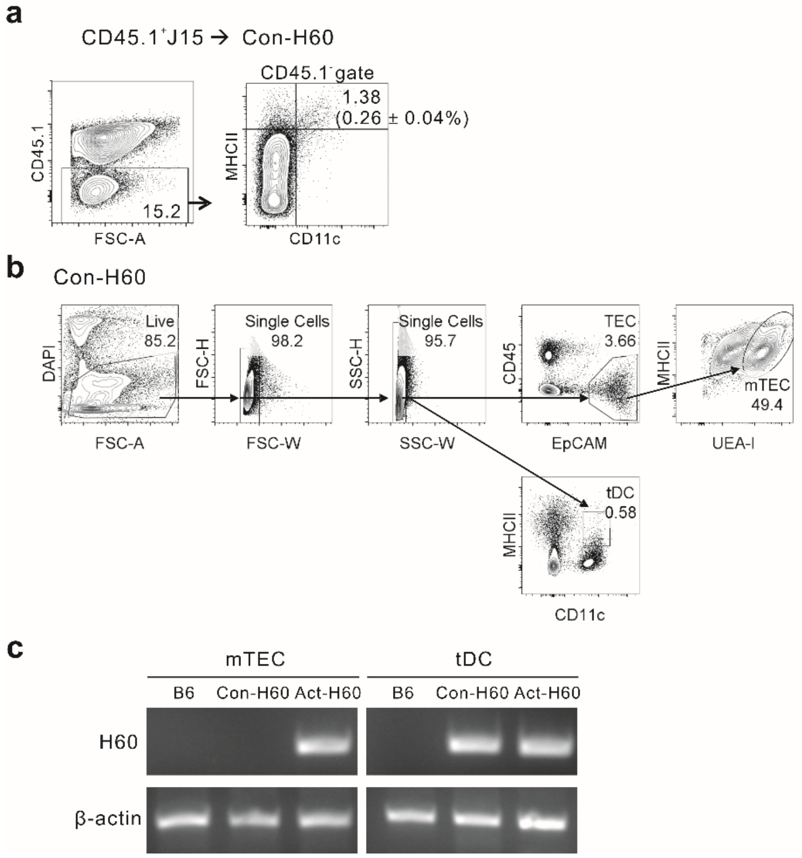

Supplementary Figure 5. Presence of host DCs in the thymus of Con-H60 recipient of CD45.1$^+$J15 BMT and their expression of H60

(**a**) Flow cytometric analysis of the thymocytes from Con-H60 recipients at 6 weeks post-CD45.1$^+$J15 BMT for detection of CD11c$^+$ MHCII$^+$ DCs of recipient origin. FACS data show the gating on the CD45.1-efluor450$^-$ cells (left) and MHCII-PE and CD11c-allophycocyanin expression in the CD45.1$^-$ cells (right). The percentages of CD45.1$^-$ cells in total thymocytes (left) and MHCII$^+$CD11c$^+$ cells in CD45.1$^-$ cells (right) are indicated. Percentages of MHCII$^+$CD11c$^+$CD45.1$^-$ cells in total thymocytes are indicated in parenthesis. Representative data from more than three independent experiments (n=3/group/experiment) are shown. Data are presented as means ± s.e.m. (**b**) Strategy for sorting of thymic DCs and mTECs from Con-H60 mice. (**c**) RT-PCR of H60 transcript in thymic DCs and mTECs sorted from Con-H60, B6, and Act-H60 mice according to the strategy shown in (**b**). β-actin expression was used as an internal control. (n=3/group/experiment). Data (**a, c**) representative of two (**c**) or at least three (**a**) independent experiments are shown.

Supplementary Figure 6

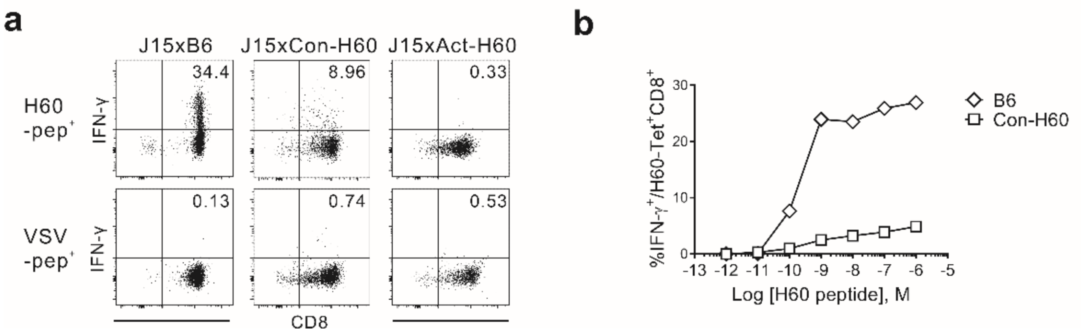

Supplementary Figure 6. TCR avidity of J15 T cells in the F1 progenies

TCR avidity assay with intracytoplasmic IFN-γ-staining**.** Splenic CD8 T cells from three different F1 progenies were stimulated *in vitro* in the presence of the indicated concentrations of H60 or VSV peptides and then subjected to intracytoplasmic anti-IFN-γ staining. (**a**) Representative FACS data of IFN-γ-APC staining of Vβ8.3^{+} CD8 T cells stimulated with 1 µM H60 or VSV peptide. (**b**) Frequencies of IFN-γ-producing cells in the H60-tet^{+}CD8^{+} cells from J15 and J15 x Con-H60 mice are plotted along the H60 peptide concentrations. (**b**) Representative FACS data show IFN-γ-APC staining of Vβ8.3^{+} CD8 T cells stimulated with 1 µM H60 or VSV peptide. Data (**a**–**b**) represent two independent experiments (n=3/group/experiment) and are presented as means ± s.e.m.

Supplementary Figure 7

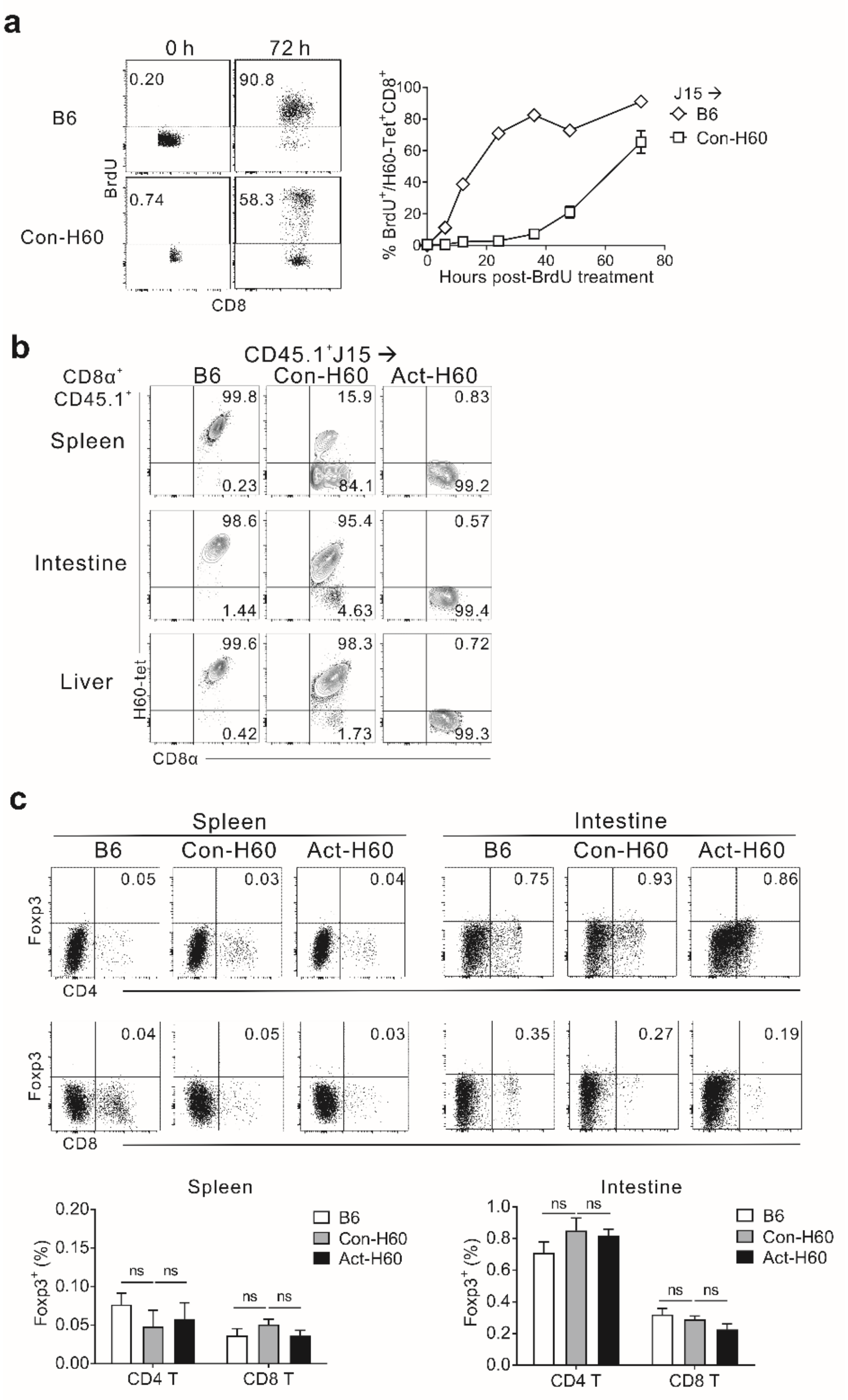

Supplementary Figure 7. Characterization of J15 escapee T cells and leukocytes in the peripheral organs of Con-H60 recipients of J15 BM.

(**a**) BrdU-incorporation during MLC of H60-tetramer^{+} splenic CD8 T cells from recipients of CD45.1^{+}J15 BM. BrdU was added to the MLC wells as described in Methods. The cells were harvested at the indicated time

points after BrdU treatment for flow cytometric analysis. Representative FACS data show BrdU incorporation by H60-tetramer$^+$ CD45.1$^+$CD8 T cells in MLC at 0 and 72 h post-BrdU treatment. Percentages of BrdU$^+$ cells in H60-tetramer$^+$ CD45.1$^+$CD8$^+$ T cells are plotted. Data represent three independent experiments (n=2/group/experiment) and are presented as means ± s.e.m. (**b**) Flow cytometric analysis of leukocytes infiltrating the spleen, intestines, and liver of recipients of CD45.1$^+$J15 BMT at 6 weeks post-BMT. Shown H60-tetramer-PE/CD8α-APC.Cy7 FACS profiles of CD45.1$^+$CD8α$^+$ cells represent more than three independent experiments (n=3/group/experiment). (**c**) Flow cytometric analysis of the proportion of Foxp3-positive T cells in the spleen and intestine of J15 BMT recipients at 6 weeks post-BMT. Representative Foxp3-PE/CD8-APC.Cy7 and CD4-PE.Cy5 FACS profiles are shown. Percentages of Foxp3$^+$ CD4 or CD8 T cells among splenocytes and intestine-infiltrating leukocytes are plotted. Data represent two independent experiments (n=3/group/experiment) and are presented as means ± s.e.m. Student's t-test was performed..

Supplementary Figure 8

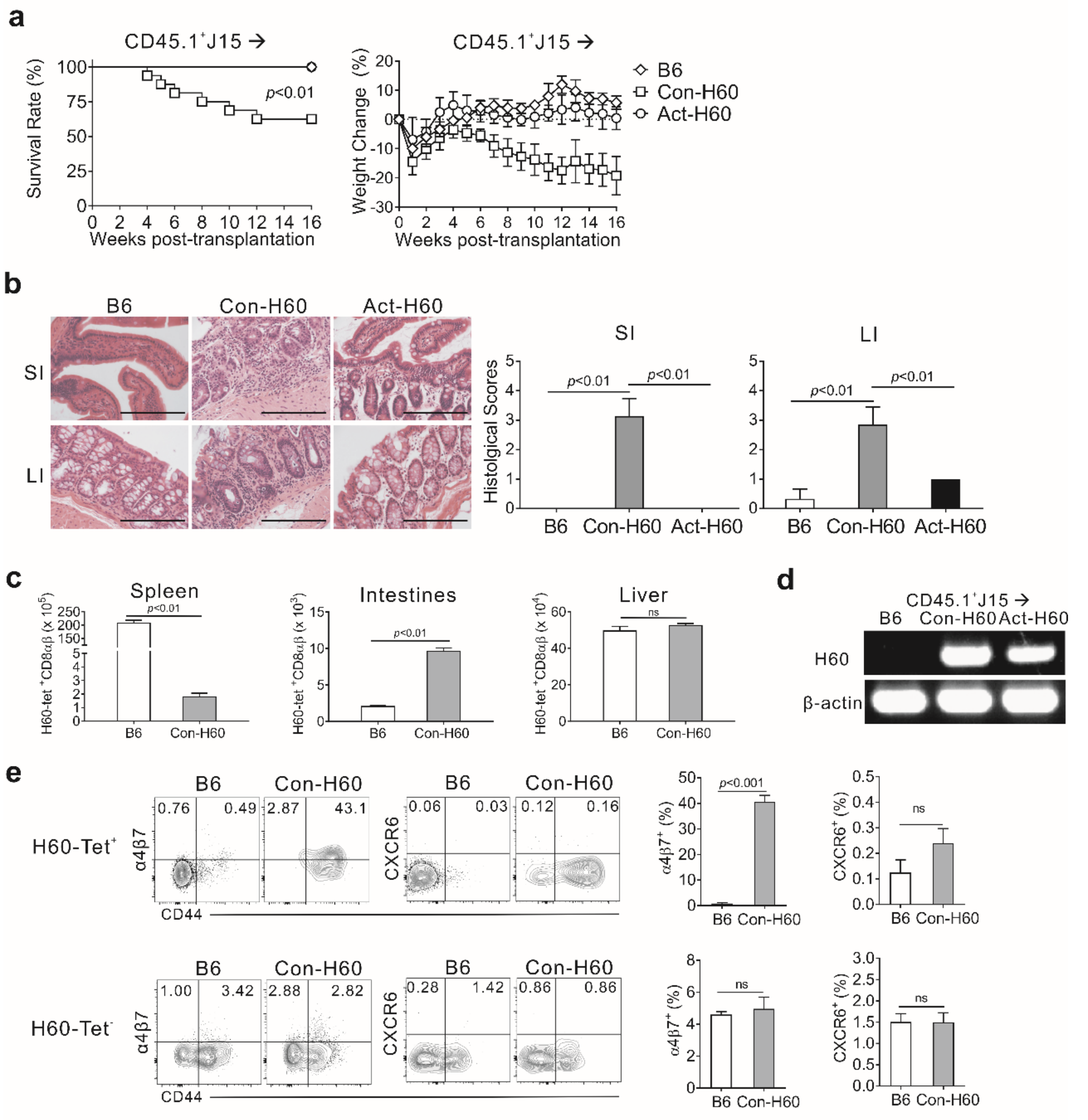

Supplementary Figure 8. Minor incidence of intestine GVHD-like pathology in Con-H60 recipients of J15 BMT

(**a**) Long-term examination of recipients of CD45.1$^+$J15 BMT. The survival rates and weight changes of the recipients are plotted. Pooled data from two independent experiments are shown (n=15 mice/group). *P* value was determined by log-rank (Mantel-Cox) test. (**b**) Histological examination of the intestines of BMT recipients. Tissue sections of the small intestine (SI) and large intestine (LI) were prepared at 8 weeks post-BMT, stained with H&E, and examined under a microscope. Three independent experiments were performed (n=2/group/experiment). Representative images of H&E-stained tissues are shown (×200, bar=100 μm). Histological scores are plotted. (**c**) Numbers of H60-tetramer$^+$ CD8αβ$^+$ T cells infiltrating the spleen, intestine, and liver of recipients of CD45.1$^+$J15 BMT at week 6 post-BMT. Data represent more than

three independent experiments (n=3/group/experiment) (**d**) RT-PCR analysis of H60 transcript in leukocytes infiltrating the intestines of J15 BM recipients at 4 weeks post-BMT. β-actin expression was used as an internal control. Data represent two independent experiments (n=3/group/experiment). (**e**) Flow cytometric analysis of the expression of the gut-homing receptor α4β7 and liver-homing receptor CXCR6 by splenic $CD45.1^{+}$CD8 T cells from J15 BM recipients. Representative α4β7-PE by CD44-FITC and CXCR6-APC by CD44-FITC FACS profiles of $H60\text{-tetramer}^{+}$ and $H60\text{-tetramer}^{-}CD45.1^{+}$ CD8 T cells are shown. Percentages of $α4β7^{+}$ cells and $CXCR6^{+}$ cells among $H60\text{-tetramer}^{+}$ and $H60\text{-tetramer}^{-}$ $CD45.1^{+}$ CD8 T cells are plotted. Data represent three independent experiments (n=3/group/experiment). Data (**b**,**c**,**e**) are presented as means ± s.e.m. *P* values were generated by Student's t-test.

Supplementary Figure 9

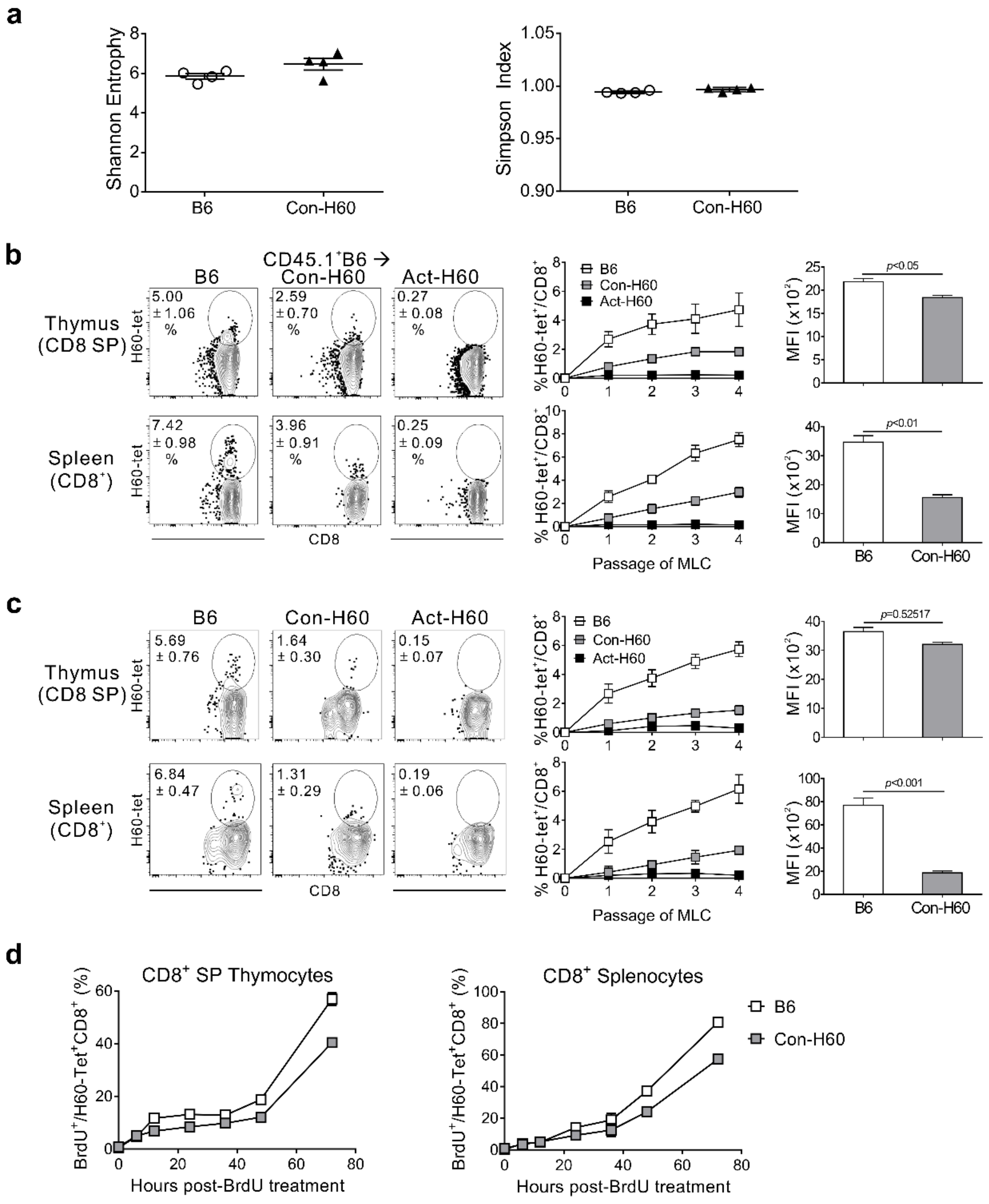

Supplementary Figure 9. H60-cognate polyclonal CD8 T cells in *in vitro* MLC cells

(**a**) Diversity of TCRβ sequences of H60-tetramer-binding splenic CD8 T cells generated in Con-H60 and B6 recipients of CD45.1$^+$ B6 BM cells. Evaluation of TCRβ sequence diversity was based on Shannon entropy and Simpson diversity scores. Scores of individual mice in each group were plotted (n=4/group). (**b–c**) H60-tetramer staining of *in vitro* cells. (**a**) H60-tetramer staining of cells in the first MLC and subsequent re-stimulation of CD8$^+$ thymocytes and splenocytes from CD45.1$^+$ BMT recipients. Cells were stained with H60-tetramer-PE, anti-CD45.1-FITC, and anti-CD8-allophycocyanin antibodies on day 5 after stimulation with the

H60$^+$ feeder cells. The percentages of H60-tetramer$^+$ cells in the CD8$^+$ *in vitro* cells and the MFI values of H60-tetramer-staining are plotted. Representative FACS data are shown after CD45.1$^+$ cell gating; the percentage values of H60-tetramer$^+$ cells are indicated. (**c**) H60-tetramer staining of *in vitro* cells originated from normal Con-H60, Act-H60, or Act-H60 mice. Data were processed as described above. Representative FACS data show the H60-tetramer staining of the *in vitro* cells after the fourth re-stimulation; the percentage values of H60-tetramer$^+$ cells are indicated. (**d**) BrdU-incorporation during MLC by H60-tetramer$^+$ thymic and splenic CD45.1$^+$ CD8 T cells from recipients of CD45.1$^+$B6 BM. Percentages of BrdU$^+$ cells among H60-tetramer$^+$ CD45.1$^+$CD8$^+$ cells are plotted. Data (**b-d**) represent three (**b, c**) and two (**d**) independent experiments (n=3/group/experiment), respectively, and are presented as means ± s.e.m. *P* values were generated by Student's t-test.
